# Mobility in community-dwelling older adults; what are its determinants?

**DOI:** 10.1186/s12877-021-02174-1

**Published:** 2021-04-07

**Authors:** Wiebren Zijlstra, Eleftheria Giannouli

**Affiliations:** Institute of Movement and Sport Gerontology, German Sport University Cologne, Am Sportpark Müngersdorf 6, 50933 Cologne, Germany

**Keywords:** Mobility, Life-space, Motion sensors, GPS, Monitoring, Capacity, Performance

## Abstract

**Background:**

Based on a conceptual framework, Kuspinar and colleagues analysed life-space mobility in community-dwelling older adults. However, a number of earlier mobility studies that used the same framework remained undiscussed. This correspondence article addresses similarities and differences between these studies, as well as highlight issues that need to be addressed to improve our understanding of mobility determinants in older adults.

**Findings:**

Despite differences in methodological approach as well as in detailed results, the studies share one important outcome: regardless of the specific choice of potential mobility determinants, only a low to moderate proportion of mobility could be explained.

**Conclusions:**

Our present understanding of the determinants of mobility in community-dwelling older adults is limited. A consistent terminology that takes into account the different aspects of mobility; the use of objective methods to assess real-life mobility; and monitoring changes in real-life mobility in response to interventions will contribute to furthering our understanding of mobility determinants.

## Background

With great interest, we read the BMC Geriatrics publication of Kuspinar and colleagues about life-space mobility in older adults [1]. The paper’s results are an important extension of early findings by Meyer et al. [2] and by other recent studies [3–6] that used similar approaches to study mobility in community-dwelling older adults. Since none of these recent studies was discussed in the paper by Kuspinar et al., this correspondence article aims to address similarities and differences with earlier studies of life-space mobility as well as highlight issues that need to be addressed to improve our understanding of mobility in older adults.

## Determinants of life-space mobility in older adults

A number of recent studies investigated how real-life mobility of community-dwelling older adults is associated to measures of physical capacity [3], cognitive capacity [4], and a combination of measures from multiple domains of functioning [5]. Another recent study [6] analysed potential determinants of life-space mobility in community-dwelling older persons with mild to moderate cognitive impairment following geriatric rehabilitation. The latter two studies (i.e. Ullrich et al. [6] and Giannouli et al. [5]) used the conceptual framework by Webber et al. [7] to identify potential mobility determinants. Both approaches included demographical and environmental variables in addition to a selection of relevant measures from four different domains of functioning; physical, cognitive, psychological, and social. Similar to studies [5, 6], Kuspinar and colleagues [1] used the Webber et al. framework to identify factors related to life-space mobility in community-dwelling older adults. However, it seems that their approach used the framework for a post-hoc analysis of existing data rather than a systematic a-priori choice of relevant domain specific measures.

Given their use of the same conceptual framework, the three studies [1, 5, 6] covered similar domains, however, their domain specific measures are different. The choice of measures from the physical domain was partly the same (i.e. all three studies included some measures of gait and/or balance; measures of muscle strength were used in [1, 5]; and body mass index was used in [1, 6]). Some similarity also exists in the psycho-social domain (e.g. all studies included measures of social support and depression (studies [5, 6] both used the ‘Geriatric Depression Scale’); and studies [5, 6] both included self-efficacy measures). Within the cognitive domain, the studies showed the largest variety; the ‘Mini Mental State Examination’ was used in study [6]; measures of set-shifting ability, and verbal learning and memory were used in study [1]; whereas planning ability, visuo-spatial attention, and spatial working memory or switching were used in study [5].

The studies used different approaches to assess life-space mobility. While Kuspinar et al. [1] and Ulrich et al. [6] assessed life-space mobility based on self-reported data (according to Peel et al. [8]), studies by Giannouli et al. [3–5] measured mobility based on objective assessments in real-life. The latter studies [3–5] assessed real-life mobility using motion sensing data (i.e. in-built accelerometers as well as position tracking based on the global positioning system (GPS)) which were obtained over seven subsequent days using a smart phone. The study into predictors of real-life mobility [5] only used three GPS based measures of life-space mobility (i.e. ‘life-space area’, ‘total distance’, and ‘maximum action range’. However, additional analyses of GPS-derived data demonstrate the possibility of in-depth analyses of different aspects of life-space mobility [9].

One major difference between the three studies [1, 5, 6] is the impressive data set that was available to Kuspinar et al. [1]; their data were collected as part of the Canadian longitudinal study on aging and comprised more than 12,500 older participants, whereas study [5] comprised more than 150, and study [6] included 118 older community-dwelling older adults. The large data set of Kuspinar and colleagues enables robust findings, and the authors are to be commended for this achievement.

Despite methodological differences, the three studies [1, 5, 6] share some remarkable outcomes in their analyses of relationships between multi-domain measures and life-space mobility in older persons. First of all, data analyses of studies [1, 5] demonstrate that only a very low proportion of life-space mobility is explained by multi-domain measures (i.e. 13.5% in study [1], and less than 15% in study [5]). Study [6] in older persons with cognitive impairment show a somewhat higher proportion of explained variance (36.3%). We think these findings are of paramount importance as they all indicate that, regardless of the choice of domain specific measures, the explanatory value for life-space mobility is low to moderate. Secondly, results of all three studies highlight that, contrary to a-priori expectations, measures of cognitive functioning did not show strong associations with life-space mobility.

Some divergence in findings can be observed when considering the strongest predictors of life-space mobility; in study [1], driving, social support and gait speed emerged as main correlates, whereas study [5] found strongest associations with the physical domain (i.e. leg and grip strength) and the psychological domain (i.e. the ‘Falls Efficacy Scale’). Study [6] found strongest associations with the physical domain (i.e. the ‘Short Physical Performance Battery’), and social activities. However, particularly given the low proportions of explained variance, these differences in study outcomes should not be overemphasized. Overall, the results of these studies indicate the need for more insight in determinants of mobility in real-life.

## Challenges to be addressed by mobility studies

Given its importance for overall health and functioning, a vast number of studies have addressed different aspects of mobility in various older populations. However, as is illustrated by the previous discussion of studies of mobility in community-dwelling older adults [1, 5, 6], our present understanding of mobility determinants is limited. Major issues that need to be addressed in order to further our understanding are the overall complexity of mobility and the manifold of methodological approaches that are in use to study mobility.

A common approach to study mobility is to use standardised tests that inform about how well a person can execute specific mobility related activities. Examples of such “capacity” tests are gait tests, sit-to-stand tests, or the ‘Timed Up and Go’ test. An underlying assumption of all of these tests is that their outcomes inform about mobility “performance” in real-life. However, recent studies [3, 10] clearly demonstrate that laboratory based measures of mobility capacity in older adults have limited value for predicting mobility performance in real-life. Such findings emphasize the need to assess mobility of older subjects in their daily environments and not only by using standardised mobility tests. However, when assessing real-life mobility, a clear distinction should be made between measures of life-space mobility, which obviously also reflect the use of transportation, and mobility measures which primarily reflect a persons’ own mobility related physical activity.

Given the complexity of real-life mobility, researchers need to explicitly focus on specific mobility aspects, adopt an appropriate methodology, and be consistent in reporting and interpreting study outcomes. Unfortunately, such is not that straightforward; results of widely different approaches have been reported without a clear terminology that allows consistent analysis of different aspects of mobility. Obviously, such prevents a systematic evaluation of mobility studies.

## Conclusions

Without claiming to be comprehensive, we think at least following issues should be taken into account in order to improve our understanding of mobility determinants in older adults:

First of all, real-life mobility should be studied based on a sound assessment approach. Commonly used methods are based on subjective reports of physical activity and/or life-space mobility. However, these require participants to report their activity, and several issues may prevent a subject from reliable reporting. Hence, subjective reports are known to be susceptible for different sources of bias. Additional disadvantages of self-reported data include limited accuracy & precision, ceiling and floor effects. Thus, subjective reports may be of limited use for assessing changes in activity in specific target groups.

A common argument for applying questionnaires or other self-report based methods rather than objective assessments is that the latter are not feasible in the context of large field studies. Though this argument may have been valid in earlier times, this no longer is true. Smartphone based studies [3–5], as well as other recent studies, demonstrate the feasibility of assessing and monitoring mobility based on wearable technology. Such an approach allows to monitor mobility related activities such as sitting, standing, walking (e.g. see [11]), and can also include more elaborate GPS based analyses which inform about life-space mobility (e.g. see [9]).

Second, future studies should comprise longitudinal designs which systematically study changes in real-life mobility in dependence of (potential) determinants rather than only use cross-sectional study designs. Though cross-sectional data can show associations (as in [1, 5, 6]), they cannot demonstrate causality. For improving our understanding of real-life mobility, we also need to study changes in mobility in response to interventions. Thus, we can study the effects of specific changes in modifiable factors, which are expected to have an impact on mobility in older persons. Such studies may require mobility monitoring approaches over long time intervals (weeks/months).

Lastly, there is an urgent need for a comprehensive mobility definition which takes into account the complexity of mobility related behaviour and which allows a consistent terminology in different mobility studies. Mobility research is a prominent topic in health sciences, and given the many aspects to mobility and the specific mobility interests of researchers and clinicians from multiple disciplines, mobility is often studied without clear and consistent definitions. This hampers our understanding of mobility, and needs be resolved by a clear and commonly accepted terminology and definitions to address mobility.

## Data Availability

Not applicable.

## Abbreviations

GPS: Global Positioning System
